# Learning spatial orientation tasks in the radial-maze and structural variation in the hippocampus in inbred mice

**DOI:** 10.1186/1744-9081-1-3

**Published:** 2005-04-22

**Authors:** Wim E Crusio, Herbert Schwegler

**Affiliations:** 1 Brudnick Neuropsychiatric Research Institute, Department of Psychiatry, University of Massachusetts Medical School, 303 Belmont Street, Worcester, MA 01604, USA; 2 Institut für Anatomie, Universität Magdeburg, Magdeburg, Germany

## Abstract

In the present paper we review a series of experiments showing that heritable variations in the size of the hippocampal intra- and infrapyramidal mossy fiber (IIPMF) terminal fields correlate with performance in spatial, but not non-spatial radial-maze tasks. Experimental manipulation of the size of this projection by means of early postnatal hyperthyroidism produces the effects predicted from the correlations obtained with inbred mouse strains. Although the physiological mechanisms behind these correlations are unknown as yet, several lines of evidence indicate that these correlations are causal.

## Introduction

Several learning tasks are available to test spatial orientation abilities in mice. The most widely applied one is probably the water navigation task (also known as the "Morris maze"), which was developed originally for rats [1]. However, it has been noted that mice are animals living in dry habitats [2,3], so that a swimming task may be less appropriate to them, because of the stress it may be expected to induce [4,5]. A factor analysis of data from several thousand mice was carried out by Wolfer and Lipp [6], who reported that only the third and least important factor showed loadings of behavioral variables related to spatial orientation, explaining less than 20% of the observed variance in behavior. Indeed, mice with hippocampal lesions still can improve their performances in this task over time [7]. This does not necessarily mean that we should abandon the Morris water navigation task for use with mice, it just means that this test may reveal differences between groups of animals that may relate to different factors and therefore should not automatically be interpreted as differences in spatial learning ability. It would therefore appear that appropriately designed dry-land mazes might assess spatial learning capacities of mice more specifically than water mazes. Among the available mazes, the radial maze appears to be especially suitable [4].

## Designing a radial maze

**Figure 1:**
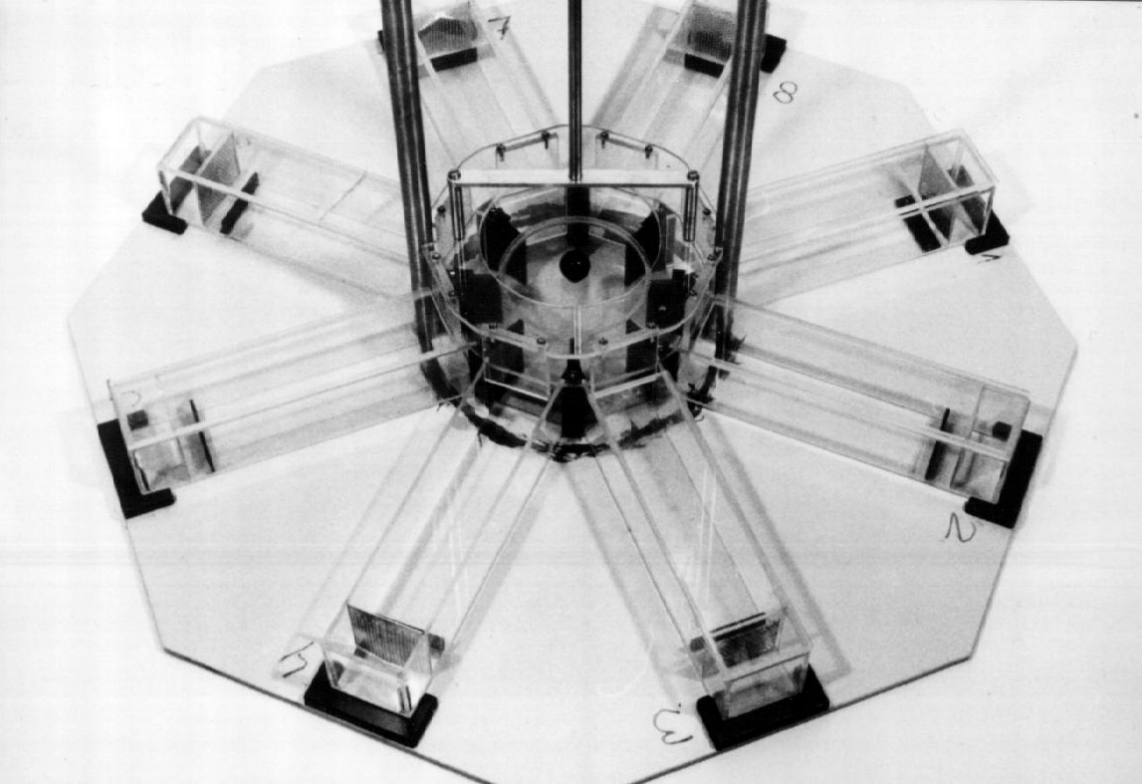
Radial maze for use with mice. Plexiglas doors can be lowered to limit access to all arms simultaneously. From Crusio, 1999 [4], with permission.

As with many behavioral tasks, the radial maze was originally developed for use with rats. It consists of a central platform, with 4–17 arms radiating outwards. The configuration that is most frequently applied uses eight arms. A food reward may be present at the end of the arms, which food-deprived subjects (maintained at 85–90% of free-feeding body weight) must locate. In our studies, we have therefore attempted to adapt this device to mice, which are generally more anxious and more sensitive to stress than laboratory rats (which have probably been selected more strongly for docility than laboratory mice). To avoid elevation-induced anxiety, the maze we use is placed on the floor of the animal room and arms are enclosed. The arms have to be transparent, to enable animals to see extramaze visual cues, without which it would indeed be very difficult for the subjects to orient themselves in space. The fact that many commercially available radial mazes have metal walls, shows that this necessary condition is perhaps less obvious than might be thought at first sight. We also reduced the dimensions of the maze relative to those habitually used with rats: the central platform measured 22 cm in diameter, arms were 25 cm long, 6 cm wide, and 6 cm high. In addition, at the end of each arm a small compartment, separated from the rest of the arms by a perforated plate, always contained fresh food in order to saturate each arm with food odors, whereas a low barrier prevented animals from seeing the possible food reward hidden behind it. During the tests, animals would therefore have to remember which food rewards had already been eaten, because this procedure made it effectively impossible to smell or see the presence of a food reward in any particular arm while the animal is still standing on the central platform (Fig. 1). Using this apparatus, we carried out a number of studies investigating possible covariation between neuroanatomical variation in the mouse hippocampus and performance in different tasks, requiring either spatial or non-spatial modes of navigation.

## Heritable neuroanatomical variation in the hippocampus

**Figure 2:**
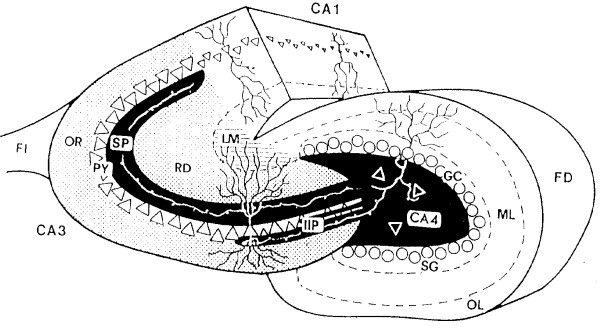
Diagram of a Timm-stained cross-section of the hippocampus. The hippocampal subregion CA3-CA4 (the area of morphometry) is indicated in black, stippled and hatched areas. Black areas: suprapyramidal (SP), intra- and infrapyramidal (IIP) and hilar (CA4) mossy fiber terminal fields originating from the dentate gyrus. Stippled area: strata oriens (OR) and radiatum (RD). Hatched area: stratum lacunosum-moleculare (LM). CA1, subregion of the hippocampus without mossy fibers; FI, fimbria hippocampi; FD, fascia dentata; OL and ML, outer and middle molecular layers of the fascia dentata; SG, supragranular layer; GC, granular cells.

Over a quarter century ago, the pioneering neurogeneticists Richard and Cynthia Wimer [8,9] described large strain differences in the distribution of the hippocampal intra- and infrapyramidal mossy fiber projection (IIPMF; Fig. 2). Subsequently, Schwegler and Lipp showed the existence of quantitative differences as well [10]: They measured the surface of the IIPMF in consecutive sections as a measure of the total volume of this projection. Expressed as a percentage of the combined surfaces of the areas CA3 and CA4, the sizes of the IIPMF projections vary between 0.8 and 4.0% [11], that is, about a four- to five-fold variation in size. It should perhaps be noted here that these size variations are not pathological in nature. Between 35 and 53% of the differences between individuals can be attributed to genetic differences between them [12,13]. Efforts are currently underway to identify some of the genes responsible for heritable differences in hippocampal neuroanatomy [14]. The idea that these variations in neuronal connectivity might have functional consequences was rather obvious and, indeed, only a few years after the Wimers' discovery, Schwegler and Lipp reported a strong correlation between IIPMF sizes and two-way active avoidance learning in mice and rats [10,15]: animals with larger IIPMF projections turned out to be poor learners in a shuttle-box task. The latter task is peculiar in the sense that, perhaps contra-intuitively, brain-damaged animals with lesions to the hippocampal formation perform better than intact animals do [16]. As such lesions generally impair spatial orientation abilities [16], we hypothesized that an opposite correlation would be found in spatial radial maze tasks. It should perhaps be noted here that other neuroanatomical features of the hippocampus also show heritable variations [12], but since no systematic correlations between these measures and behavior have been found, we concentrate here on the IIPMF

## Simple tasks with all arms baited

**Figure 3:**
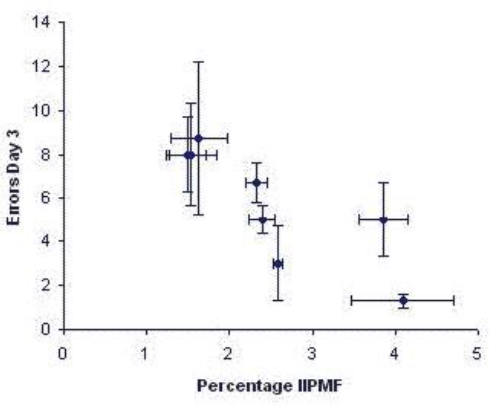
Means ± SEM of the sizes of the hippocampal intra- and infrapyramidal mossy fiber projection (IIPMF) and the numbers of errors committed in a simple radial-maze task, with free access to arms and all 8 arms containing a food reward, each dot representing the mean of one inbred strain. Hippocampal data are based on 4 male mice per strain, behavioral data are from 3 males per strain. Data taken from Crusio et al., 1987 [11].

When we started our studies, only little information about radial-maze learning abilities in mice was available and one of the very few studies available reported an inability of mice to master this task [17]. We therefore decided to carry out a pilot experiment [11], using three males from each one of eight different inbred strains, and training them on the simplest task possible. In this first study, opaque PVC arms with clear covers were used, as at the time was being done in almost all maze studies. In order to orient themselves in space, animals would therefore have to look upward. To our own surprise, it turned out that mice learned this task extremely rapidly. Applying one trial per day, animals from the fastest learning strain, C3H/HeJ made in the mean only one error (defined as repeated entrances in a previously visited arm) in the third and last training session [11]. Because only few animals per strain were used in this pilot experiment, standard errors were quite large. Nevertheless, over the eight strains investigated, the numbers of errors committed correlated strongly with the IIPMF sizes (*r*_*S *_= -0.88, df = 6, *P *< 0.01; see Fig. 3).

At first sight, this result seemed to confirm our hypothesis: animals with larger IIPMF projections committed fewer errors, mastering the task more rapidly than animals with smaller IIPMF. However, matters were perhaps more complicated than that. Upon closer examination, it appeared that many animals used a kinesthetic strategy to solve the task, visiting adjacent arms in a clockwise or counter-clockwise fashion [11]. Whether such a strategy is based on spatial orientation capabilities or not, is not directly evident. Therefore, we decided to modify the radial maze task, as it was known from work with rats that confining subjects for 5 sec to the central platform in between subsequent arm choices interrupts this kind of chaining response [18]. In addition, observations made during behavioral testing suggested that mice are probably not using extra-maze cues when opaque arms are used as they rarely seemed to look upwards. We therefore replaced the PVC arms with arms made of clear Plexiglas and installed guillotine doors to enable the application of a confinement procedure. To facilitate the use of extra-maze cues for spatial orientation, we placed several objects close to the maze (of course, the experimentator is already a very visible cue in him/herself). This was done because the best-performing strain in the pilot experiment, C3H/HeJ, carries the *Pde6b*^*rd*1 ^mutation causing retinal degeneration [19]. Although these animals are not yet blind at the age that we use them (about 3 months, see [20-22]), their visual acuity obviously will be severely impaired, so we wanted to make this task as easy on their visual systems as possible.

**Figure 4:**
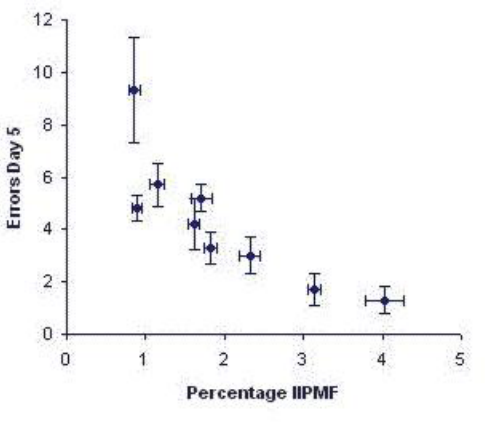
Means ± SEM of the sizes of the hippocampal intra- and infrapyramidal mossy fiber projection (IIPMF) and the numbers of errors committed in a simple radial-maze task, with subjects confined to the central platform for 5 sec. in between subsequent arm choices and all 8 arms containing a food reward. Each dot represents the mean of one inbred strain. *n *= 6 male mice per strain. Data taken from Schwegler et al., 1990 [23].

Six male mice from each one of nine different inbred strains were tested in this modified task. Because this task was expected to be more difficult than the previous one, we tested the animals for 5 days, again giving just one trial per day [23]. As in the previous experiment, animals from several strains mastered the task with surprising ease, whereas other strains did not improve their scores at all. Again, the IIPMF sizes correlated strongly with performance (*r*_*S *_= -0.92, df = 7, *P *< 0.01; see Fig. 4). However, in contrast to the previous experiment, animals did not exhibit any obvious kinesthetic strategies any more.

At the same time, we tested the same number of animals and strains in another radial-maze task that did not require any spatial orientation in order to be solved [23]. Here, opaque PVC arms were used and instead of manually operated guillotine doors we employed perforated aluminum plates fixed to the floor with adhesive tape. Subjects could easily open the doors, but as this took a few seconds, this procedure was also expected to disrupt any kinesthetic strategies. As with the previous spatial task, performance in this experiment ranged widely between different inbred strains and kinesthetic strategies were, indeed, absent. However, no correlation whatsoever with hippocampal mossy fibers became apparent (data not shown, see [23]).

The results obtained were in accordance with our hypothesis that sizes of the IIPMF would correlate with spatial learning capacities, but not with nonspatial learning abilities. These data therefore provided support for the cognitive mapping theory of O'Keefe and Nadel [16], which postulates that the hippocampus is uniquely involved in the regulation of spatial, allocentric memory. However, an alternative explanation was available, too. Olton [24] has hypothesized that the hippocampus regulates working memory, as opposed to other brain systems that would modulate reference memory, regardless of whether the information concerned was spatial or nonspatial in nature. Under this hypothesis, working memory stores information that is pertinent to one trial only (for instance, which arms have already been visited), but which has to be erased before the next trial to allow correct performance. Reference memory concerns information that is pertinent to all trials (for instance, the fact that food can be found at the end or an arm). Obviously, our spatial task had been a working memory task, whereas the nonspatial task was a reference memory task. Our results were therefore compatible with both competing theories, that of O'Keefe and Nadel [16] and that of Olton [24]. We therefore modified our task yet again, to allow simultaneous measurement of working and reference memory in both spatial and nonspatial versions of the radial maze.

## More complex tasks dissociating working and reference memory

Following Nadel and McDonald [25], we trained animals from the same nine inbred strains on a task in which only four out of the eight arms were systematically rewarded, the other four arms never containing any accessible food [26]. Two experiments were done. In one the task was spatial, using the radial maze with Plexiglas arms and guillotine doors as described above. In the other one, the task to be mastered was non-spatial, using the radial maze with opaque PVC arms, combined with guillotine doors. In both tasks, animals were confined to the central platform for 5 sec between subsequent arms choices. In the spatial version, mice were trained to locate four food rewards that were always placed in the same set of four arms. Each individual mouse had its own set of four rewarded arms. Following Olton's definition, entries into an arm that is never baited constitute a reference memory (RM) error, whereas repeat entries into an arm that has been visited previously constitute working memory (WM) errors. To prevent animals from using within-maze cues, the maze was rotated 45° at the end of each day (between subsequent trials), so that intra-maze and extra-maze cues were dissociated. This procedure prevented animals, e.g., from following hypothetical olfactory trails and forced them to use extramaze cues exclusively. In the nonspatial version, arms were marked by different black-white patterns on their floors. Food rewards were now associated with different sets of black/white patterns, each individual mouse again having its own combination of rewarded and non-rewarded patterns. As in the spatial task, RM and WM errors can now be defined.

**Figure 5:**
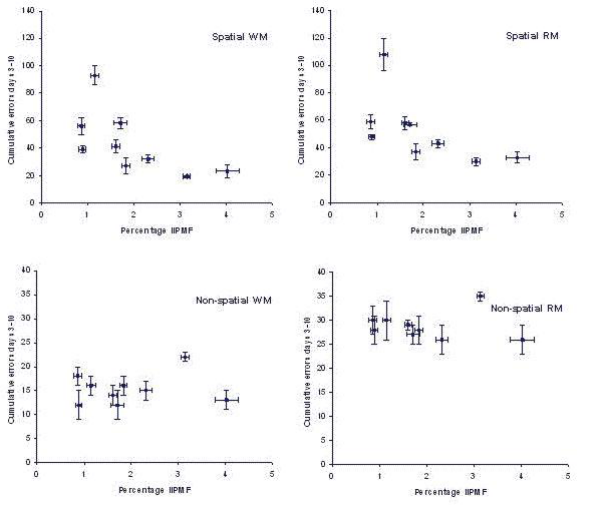
Means ± SEM of the sizes of the hippocampal intra- and infrapyramidal mossy fiber projection (IIPMF) and the numbers of working-memory (WM) and reference memory (RM) errors committed in an 8-arm radial maze with only 4 arms containing a food reward. Animals were tested during 10 days, one trial per day. Numvbers of errors shown are cumulative error counts on days 3–10. Upper panels: Spatial task. Lower panels: Non-spatial task. Left panels: Working-memory errors. Right panels: Reference memory errors. Note the different scales in the upper and lower panels, the non-spatial task obviously being much easier for the subjects.

This experiment permitted the simultaneous measurement of WM and RM errors in tasks that either required spatial orientation abilities or not. O'Keefe and Nadel's theory [16] would predict covariations between the IIPMF and both WM and RM performance in the spatial, but not in the non-spatial task. Olton's hypothesis [24] would predict covariations with WM in both the spatial and the nonspatial tasks, but not with RM in either task. As shown in Fig. 5, the results of this experiment were in complete agreement with the predictions of the cognitive mapping theory of O'Keefe and Nadel. In addition, we found that both in the spatial and in the non-spatial tasks WM and RM were correlated very strongly, raising doubt as to whether the distinction between these two types of memory really is pertinent, at least for mice. Indeed, in those experiments where authors reported a dissociation between these two forms of memory (e.g., [27]), almost invariably two different tasks were used, one purported to be a WM task, the other one an RM task. Obviously, such different tasks differ for many more components, which might explain any dissociation at least as well.

## Using the radial maze to demonstrate mutational and pharmacological effects

**Figure 6:**
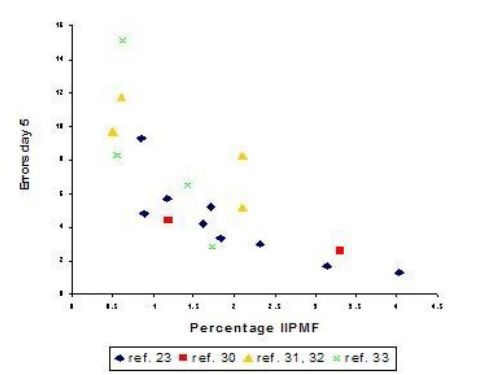
Means ± SEM of the sizes of the hippocampal intra- and infrapyramidal mossy fiber projection (IIPMF) and the numbers of errors committed in a simple radial-maze task, with free access to arms and all 8 arms containing a food reward, each dot representing the mean of one inbred strain. Data taken from Figure 4 (8 strain means) and from Refs. [30-33] (10 additional strain means). For clarity, SEMs have been omitted.

Several other authors have also investigated strain differences in radial-maze learning tasks. They reported results that were sometimes rather different from ours. However, the experimental design and apparatus used differed strongly from ours, too. For instance, Roullet and Lassalle [28] used female instead of male mice, whereas their maze and the one used by Ammassari-Teule et al. [29] was elevated. Not surprisingly, given the different behavioral results, Roullet and Lassalle [28] did not find any correlations with the IIPMF. In contrast, we performed several experiments in the spatial task with all arms baited, investigating mutational [30], Y-chromosomal [31,32], and mtDNA [33] effects on IIPMF distributions and learning behavior. When the results of all these studies are combined with those from our 1990 experiment ([23], see Fig. 6), we obtain a remarkably consistent picture. Despite the fact that these experiments took place over a period of about 10 years and were carried out by different people in different laboratories using different morphometrical methods, the overall correlation obtained is *r*_*S *_= 0.81 (df = 17, *P *< 0.0001), which is only marginally lower than the correlation found in our 1990 study [23].

Of course, correlations between two variables need not indicate a causal relationship and the IIPMF-spatial learning correlation might be spurious. Hypothetically, a third, as yet unknown, neuronal variable might be the one causing the observed strain differences in learning. The IIPMF-learning correlation would then only appear because this hypothetical third variable itself would be correlated with the IIPMF. However, in the present case we believe that there are strong indications that this correlation is, indeed, causal. First, there is the remarkable consistency and strength of the correlations reported. If a third variable would be directly correlated with learning performance, the IIPMF correlation would be only secondary and the third variable would have to correlate with learning even stronger than the IIPMF do. This would be difficult to imagine. Second, a correlation between strain means differs in one important respect from ordinary correlations, estimated from individual values. Namely, such a correlation represents a genetic correlation, meaning that gene effects on one variable are correlated with gene effects on the other variable [34,35]. Such a situation makes it highly likely that the statistical relationship found is, indeed, a causal one. Finally, we have also addressed this question in a pair of experiments in which newborn pups of a strain (DBA/2) known to possess scant IIPMF projections and feeble learning capacities in the radial maze were treated with thyroxin in the early postnatal period [36-38]. This treatment induces an increase in the size of the IIPMF in adults and we found that this increase was accompanied with a significant improvement in the spatial learning capabilities of these animals, both in a task in which all arms were baited as well as in a task in which only 4 of the arms were consistently baited.

## Other behaviors correlated with hippocampal neuroanatomy

Although the present review concerns radial-maze learning, we would like to briefly mention some other behaviors that have been found to correlate with the IIPMF. The very first correlation that was reported, with two-way active avoidance learning, has already been mentioned above (for a review, see also [39]. Other correlations that have been found are with intermale aggression [40-42], paw preference [43], reversal learning in a water navigation task [44,45], visual and tactile discrimination in a Y maze [46], and exploration [47-49] and habituation [50] in an open field.

## Conclusion

Taken together, we conclude that in inbred mice the hippocampal intra- and infrapyramidal mossy fiber projection plays an important role in the regulation and/or modulation of spatial orientation capabilities in the radial maze. Larger IIPMF projections go with better learning capabilities in the spatial radial-maze tasks described above. These correlations are specific to spatial learning, as no correlations were found in radial-maze tasks that could be solved using non-spatial cues or that employed elevated mazes, possibly inducing increased levels of anxiety in the experimental subjects.

At this point it is not yet fully understood why variations in the size of the IIPMF have such drastic consequences for an animal's behavior. It has been shown that these variations are associated with differences in spontaneous bursting in region CA3 [51] and with differences in LTP [52]. LTP is generally regarded as the most promising physiological mechanism underlying learning and memory, although the extent of its implication in these processes remains controversial (see [53] for a critical discussion). However, it was recently found that blocking of mossy fiber LTP or LTD does not abolish spatial learning capabilities in mice [54]. Therefore, the IIPMF apparently modulate behavior by other mechanisms. One possibility may be that larger IIPMF would somehow facilitate LTP in the hippocampal CA1 region. This hypothesis would be consistent with our findings that larger IIPMF go with better spatial learning abilities and diminished two-way active avoidance learning, behaviors that are abolished, respectively enhanced, after a hippocampal lesion [16]. Further research is clearly needed to address these questions.

Finally, among the different hippocampus-dependent radial maze tasks presented above, the simple spatial one (all eight arms rewarded, short confinement to the central platform between subsequent arm choices) appears to be the most useful task: It is rapid (in our protocol we use 5 daily trials only, each trial taking on a few minutes near the end of training) and we have used it successfully to investigate effects of pharmacological treatments [55,56] or to detect subtle mutational effects [57].

## Competing interests

The author(s) declare that they have no competing interests.

## Authors' contributions

The ideas presented in this manuscript are the results of many years of discussions between WEC and HS. The manuscript was drafted by WEC. Both authors read and approved the final manuscript.
